# Supplementary material for: Ruthenium anchored on carbon nanotube electrocatalyst for hydrogen production with enhanced Faradaic efficiency
Source: Nat Commun. 2020 Mar 9;11:1278. doi: 10.1038/s41467-020-15069-3 (PMC7062887; doi:10.1038/s41467-020-15069-3)
Supplement: Supplementary file 1 — Supplementary Information [file 41467_2020_15069_MOESM1_ESM.pdf]

Supplementary Information

Ruthenium anchored on carbon nanotube electrocatalyst for hydrogen production with enhanced Faradaic efficiency

By Kweon *at al.*

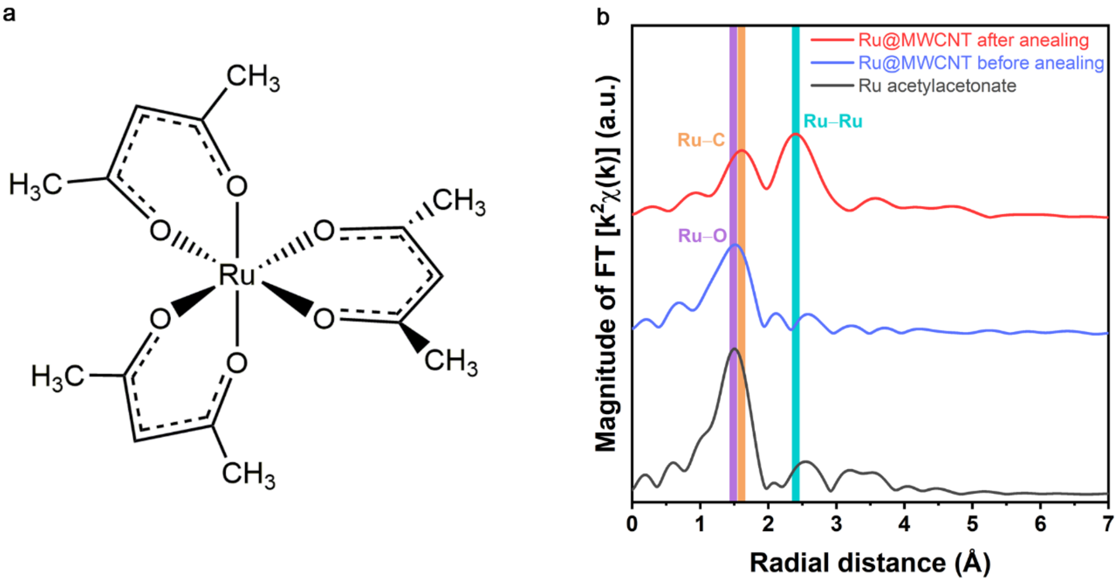

Supplementary Fig. 1 | a, Chemical structure of ruthenium acetylacetonate. **b,** Fourier transformed (FT) Ru K-edge EXAFS spectra of before/after heat-treated Ru@MWCNT and reference Ru acetylacetonate.

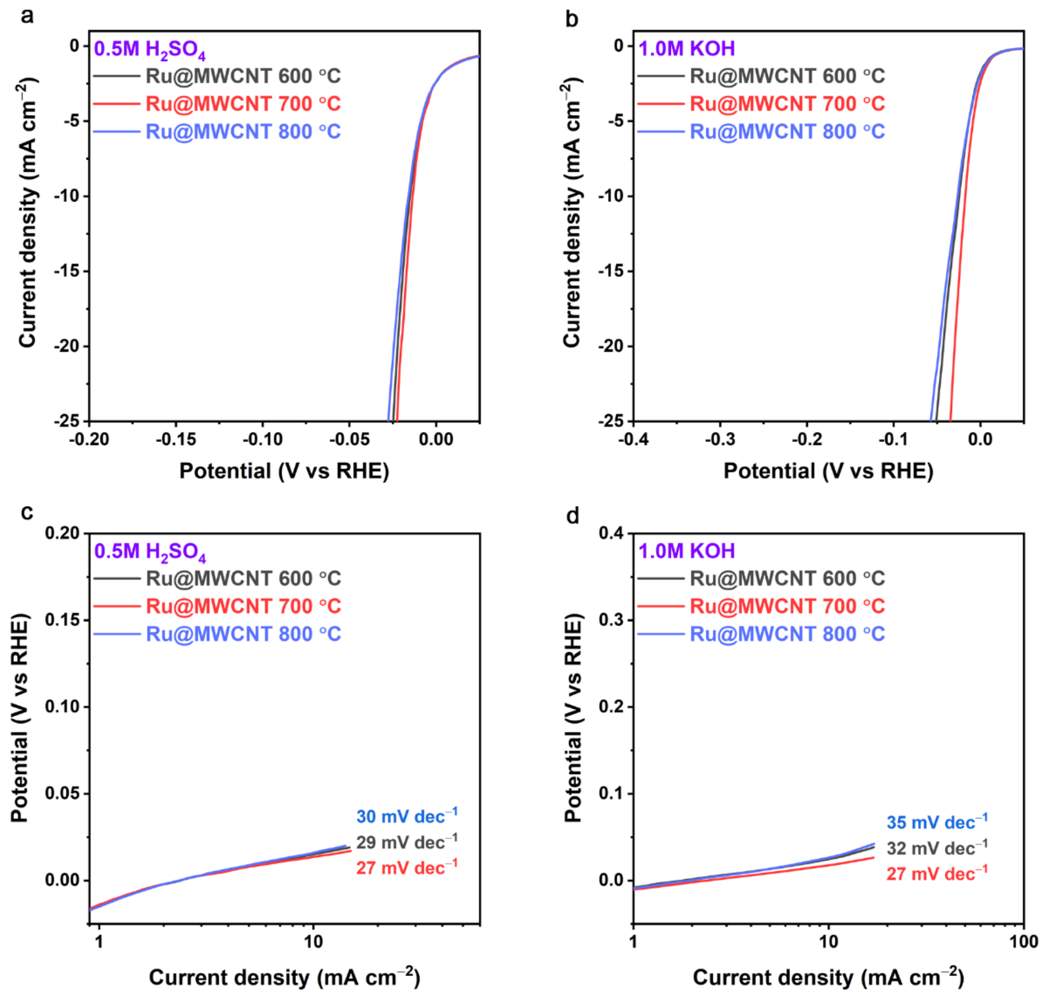

Supplementary Fig. 2 | Polarization curves of the Ru@MWCNT catalysts prepared at different annealing temperatures of 600, 700 and 800 °C: **a,** in 0.5 M aq. H_2SO_4 solution; **b,** in 1.0 M aq. KOH solution. Corresponding Tafel slopes: **c,** in 0.5 M aq. H_2SO_4 solution; **d,** in 1.0 M aq. KOH solution.

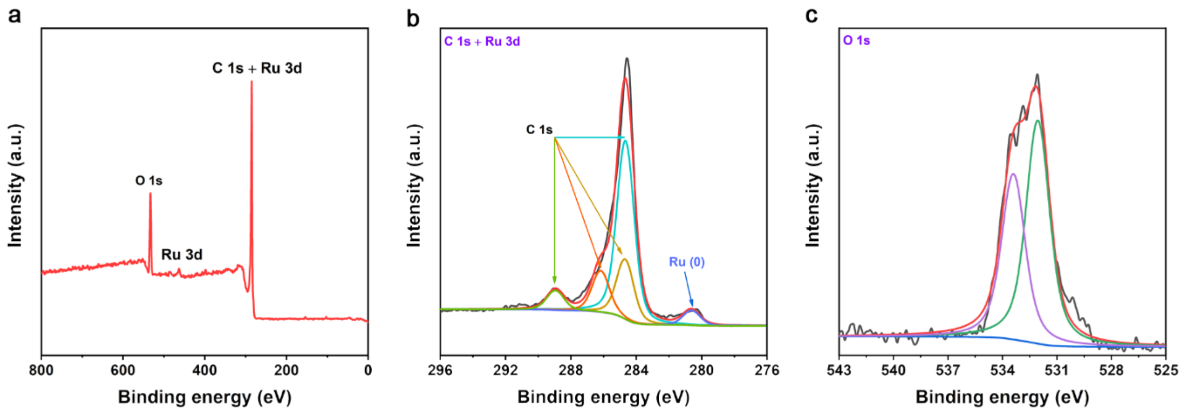

Supplementary Fig. 3 | XPS survey spectra of Ru@MWCNT after heat-treatment at 700 °C for 2h: **a,** Full survey spectrum. High-resolution spectra: **b,** C 1s and Ru 3d; **c,** O 1s.

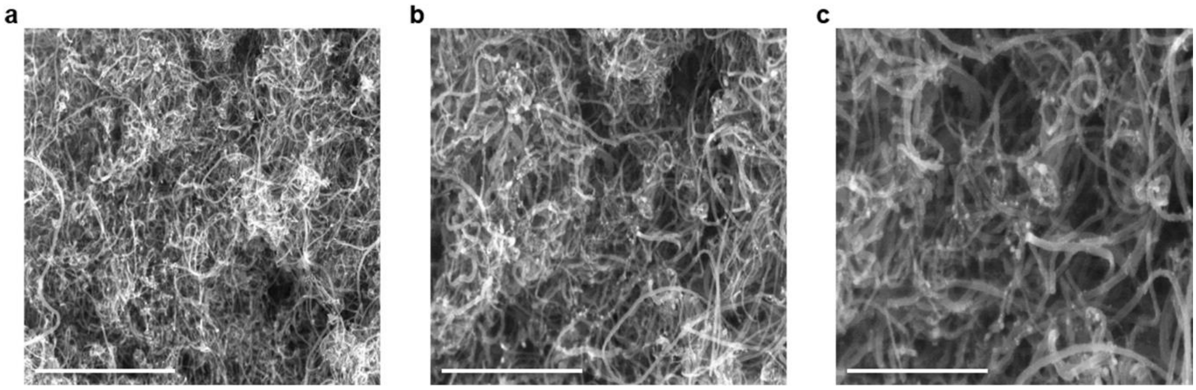

Supplementary Fig. 4 | Scanning electron microscopy (SEM) images of Ru@MWCNT at different magnifications: **a,** ×25,000; **b,** ×50,000; **c,** ×100,000. Scale bar: **a** 2 μm; **b** 1 μm; **c** 500 nm

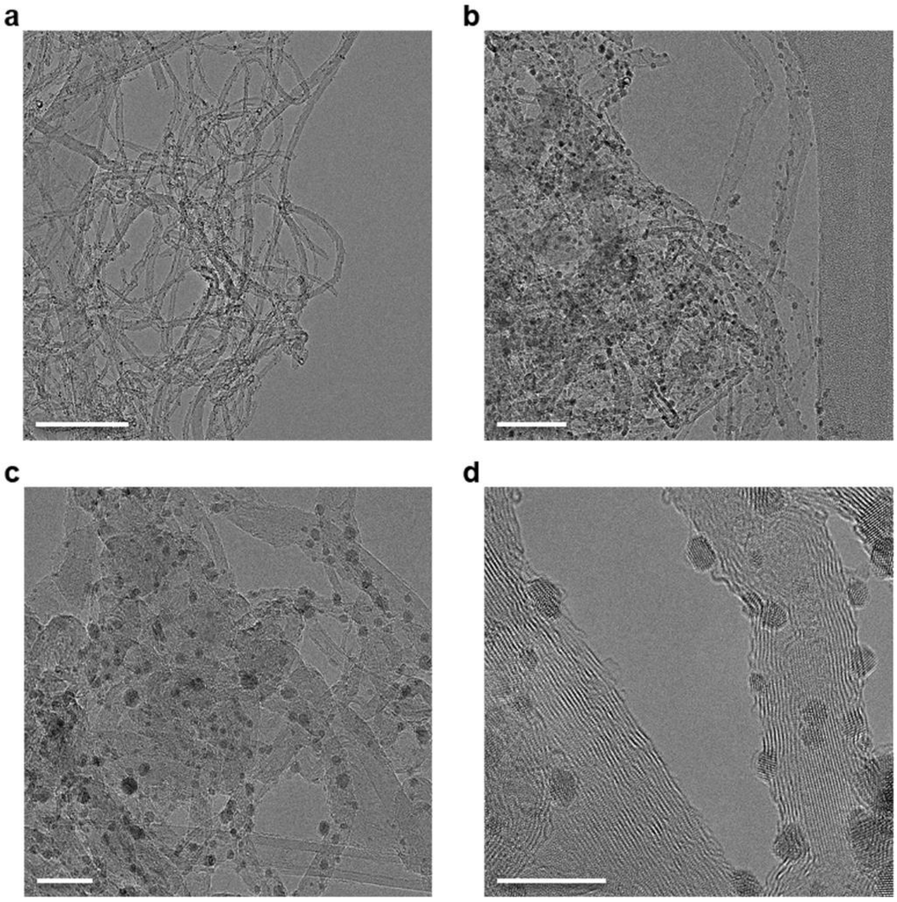

Supplementary Fig. 5 | TEM images of the Ru@MWCNT: **a-c,** Normal TEM image at different magnifications. **d,** High resolution TEM image. Scale bar: **a** 200 nm; **b** 50 nm; **c** 20 nm; **d** 10 nm

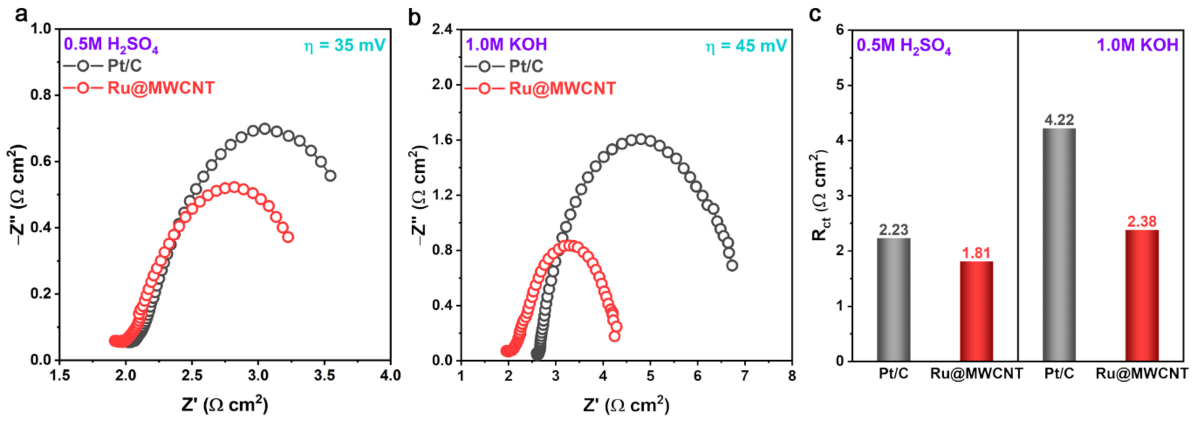

Supplementary Fig. 6 | Comparison of electrochemical impedance spectroscopy (EIS) curves of the Ru@MWCNT and Pt/C catalysts: **a,** in acidic condition; **b,** in alkaline condition. **c,** Corresponding calculated charge transfer resistances (R_{ct}) in 0.5 M aq. H_2SO_4 and 1.0 M aq. KOH solutions.

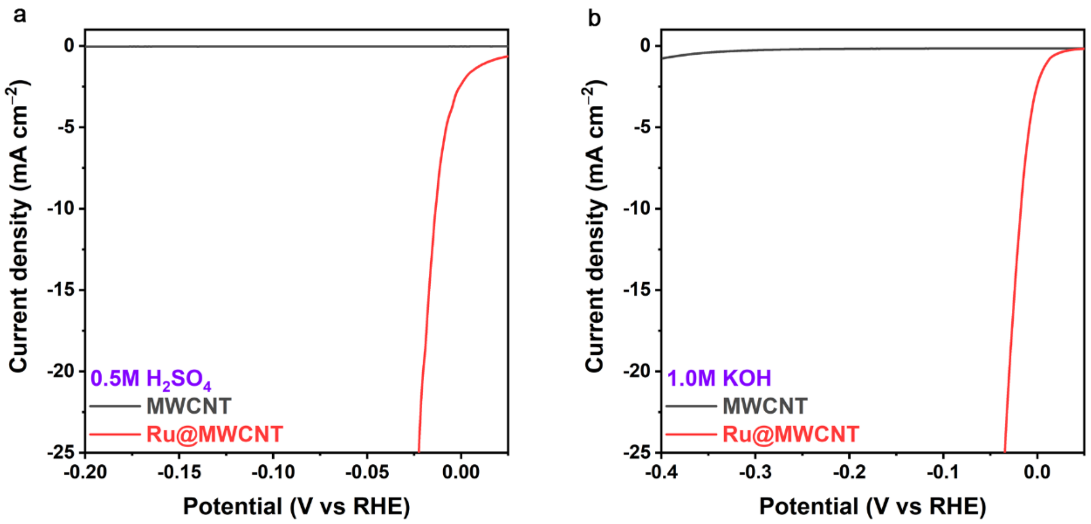

Supplementary Fig. 7 | Polarization curves of MWCNT and the Ru@MWCNT catalysts at scan rate of 5 mV s^{-1}: **a,** in 0.5 M aq. H_2SO_4 solution; **b,** in 1.0 M aq. KOH solution. As shown in the figures above, by itself the catalyst support, MWCNT, has almost negligible catalytic activity for HER even at an overpotential of 0.4 V in both acidic and alkaline conditions.

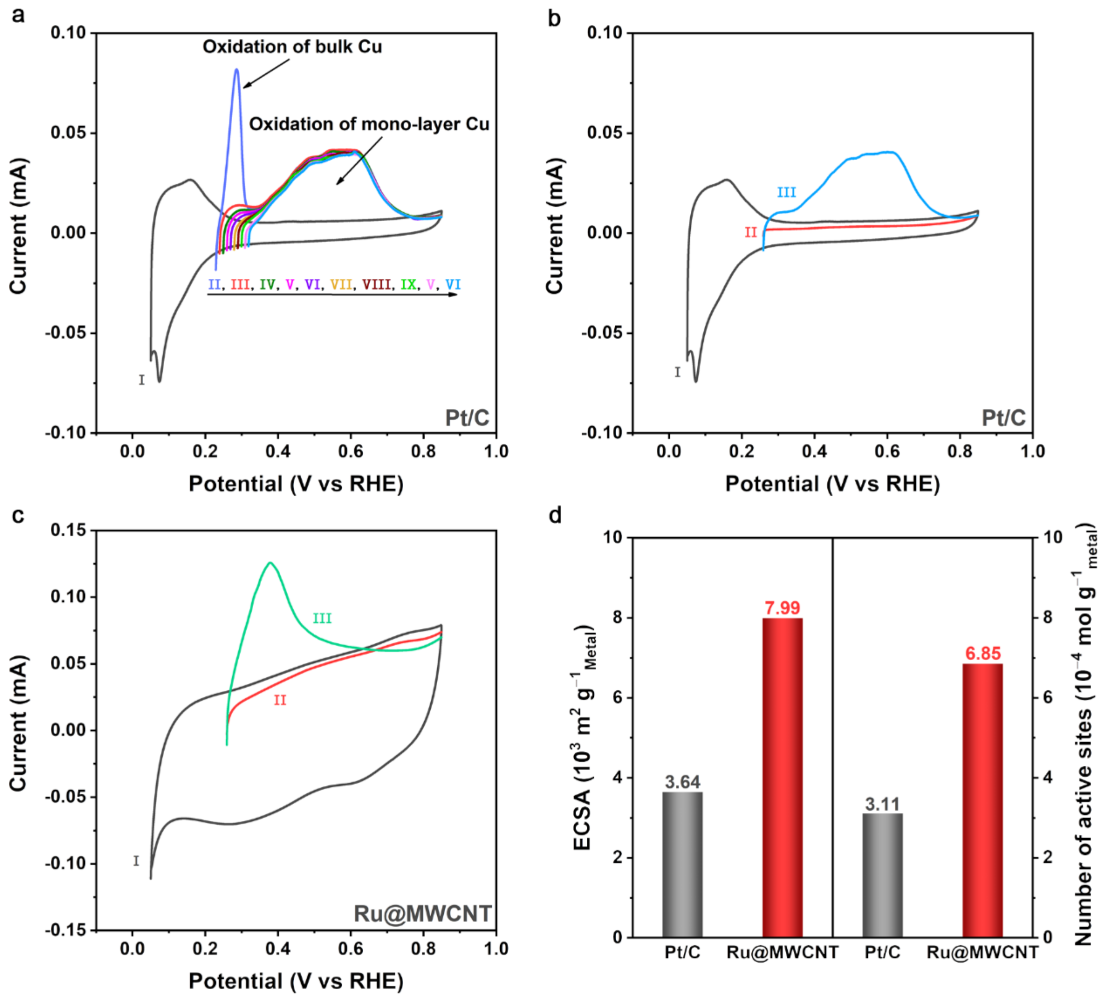

Supplementary Fig. 8 | a, Copper UPD curves in 0.5 M aq. H_2SO_4 in the absence and presence of 5 mM $CuSO_4$ on Pt/C. The electrode was polarized at 0.23 V for 100 s to form the UPD layer. **b,** Copper UPD in 0.5 M aq. H_2SO_4 in the absence and presence of 5 mM $CuSO_4$ on Pt/C. The electrode was polarized at 0.26 V for 100 s to form the UPD layer. **c**, Copper UPD in 0.5 M aq. H_2SO_4 in the absence and presence of 5 mM $CuSO_4$ on Ru@MWCNT. The electrode was polarized at 0.26 V for 100 s to form the UPD layer. **d,** Estimation of the ECSA and active sites using the Cu-UPD method.

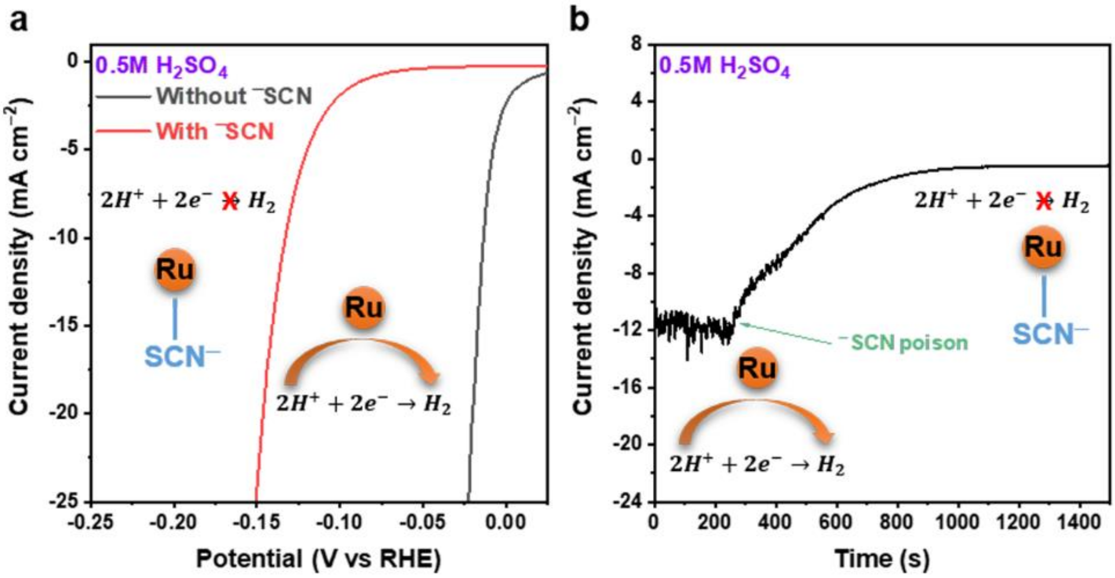

Supplementary Fig. 9 | a, Linear sweep voltammetry (LSV) curves of Ru@MWCNT before and after adding ⁻SCN ions to the 0.5 M aq. H_2SO_4 solution. **b,** Chronoampherometry (CA) curve of Ru@MWCNT before and after the addition of ⁻SCN ions to the 0.5 M aq. H_2SO_4 solution. The insets in (**a**) and (**b**) illustrate the HER pathway with/without ⁻SCN.

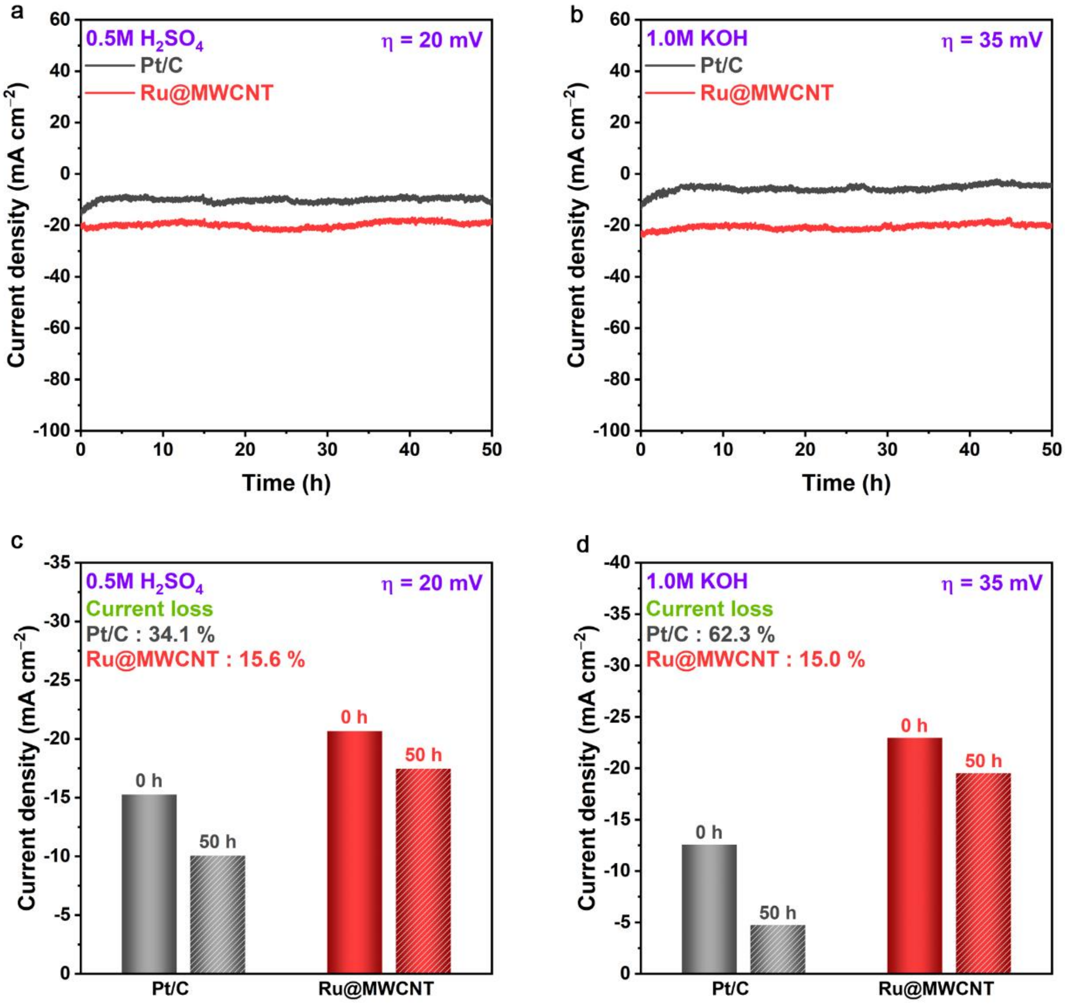

Supplementary Fig. 10 | a, b, Current-time (I *vs.* t) stability curves up to 50 h duration were recorded in 0.5M aq. H_2SO_4 and 1.0 M aq. KOH solutions. The slight current loss can be attributed to the peeling of the catalysts during the H_2 bubbles release process. **c, d,** The losses in current densities were compared between the initial point and after 50 h.

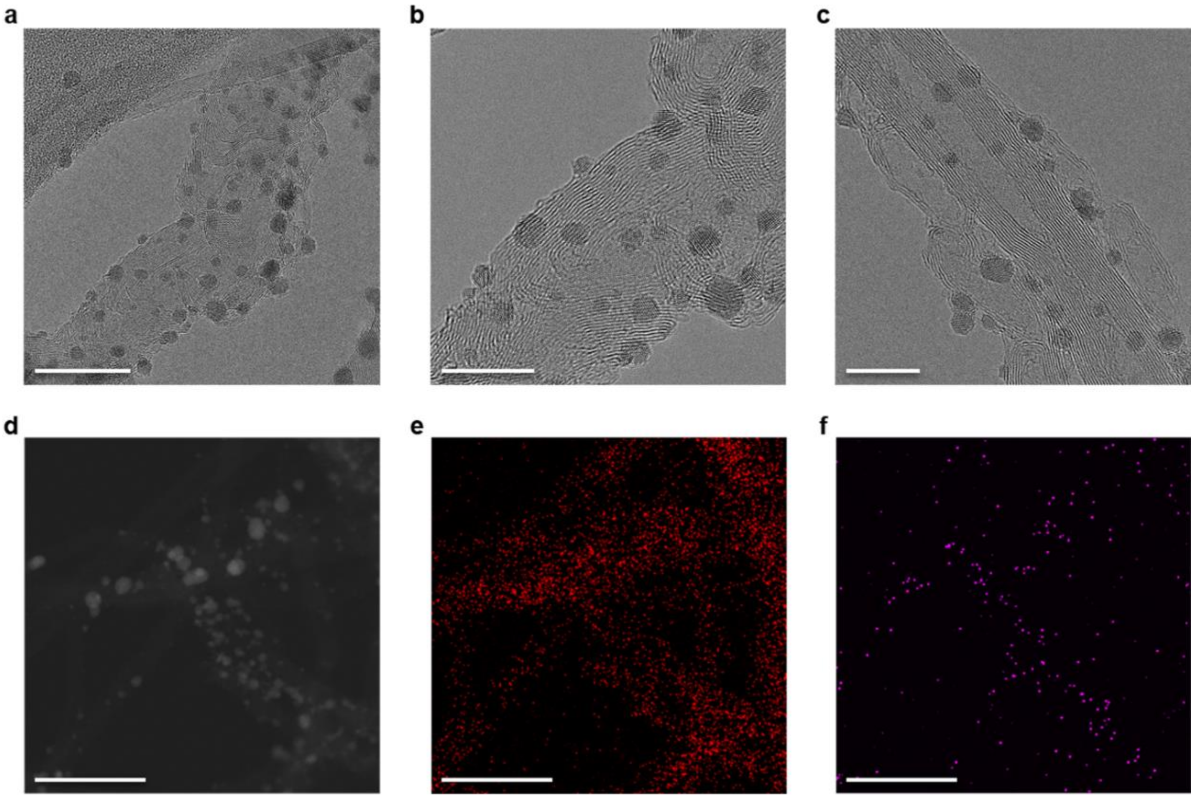

Supplementary Fig. 11 | a-c, TEM images of the Ru@MWCNT after stability testing. **d,** High-angle annular dark-field scanning transmission electron microscope (HAADF-STEM) image. **e, f,** Corresponding elemental mapping images of (**d**): **e,** carbon; **f,** ruthenium. Scale bar: **a** 20 nm; **b** 10 nm; **c** 10 nm; **d-f** 50 nm

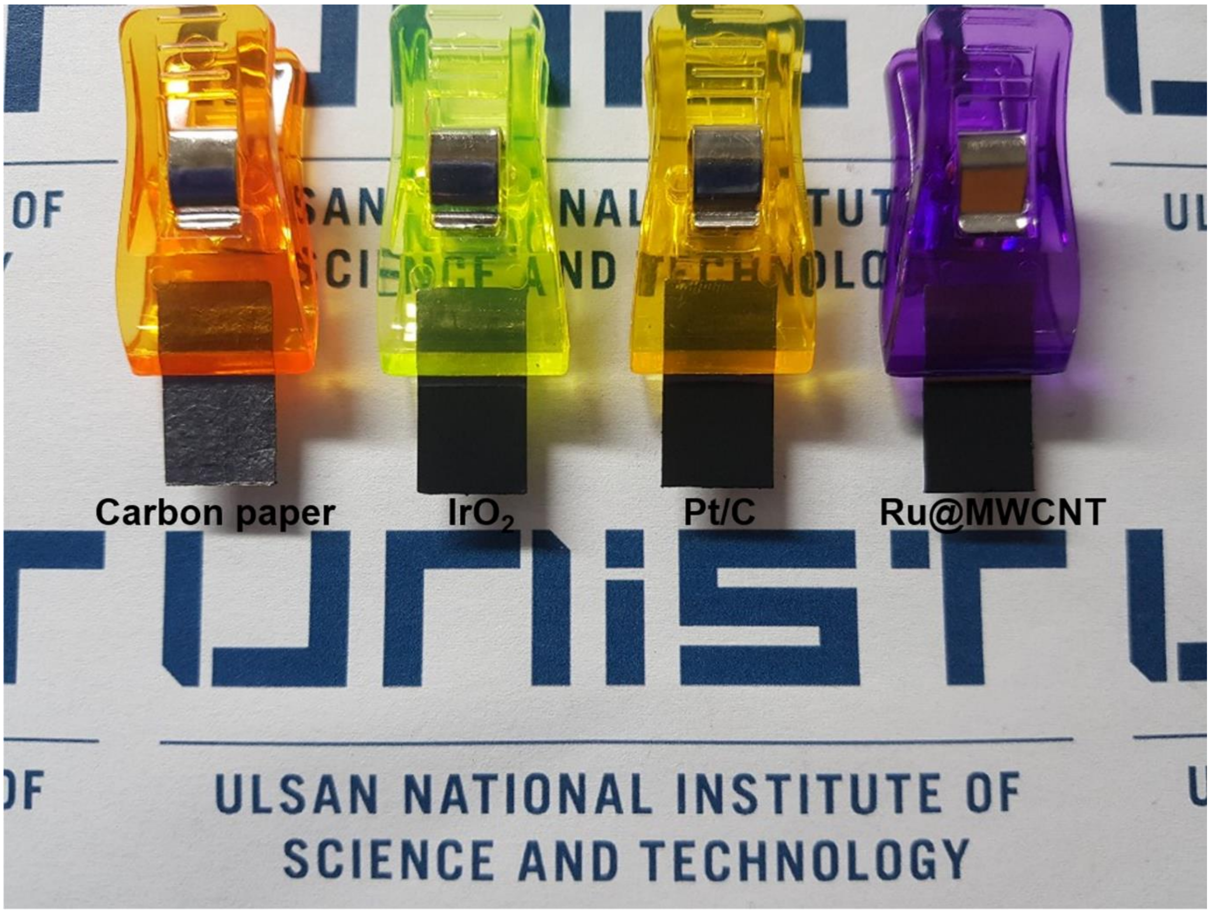

Supplementary Fig. 12 | Digital photograph of the electrodes

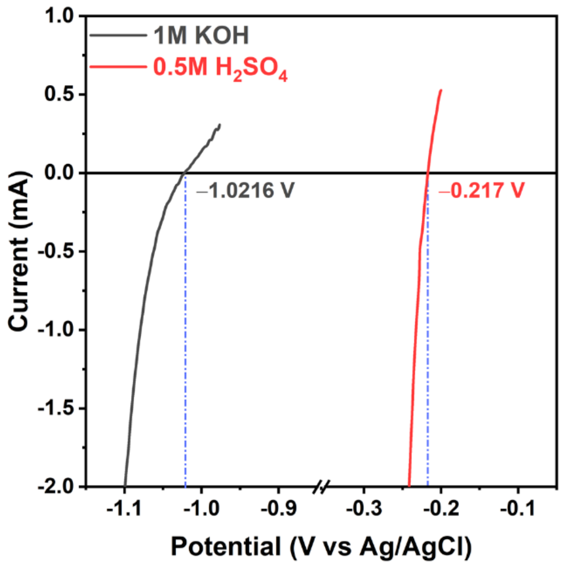

Supplementary Fig. 13 | Current-potential curves of Pt wire in highly pure H_2-saturated 0.5 M aq. H_2SO_4 and 1.0 M aq. KOH solutions, used for calibrating the Ag/AgCl electrode with respect to RHE. Scan rate: 1 mV s^{-1}.

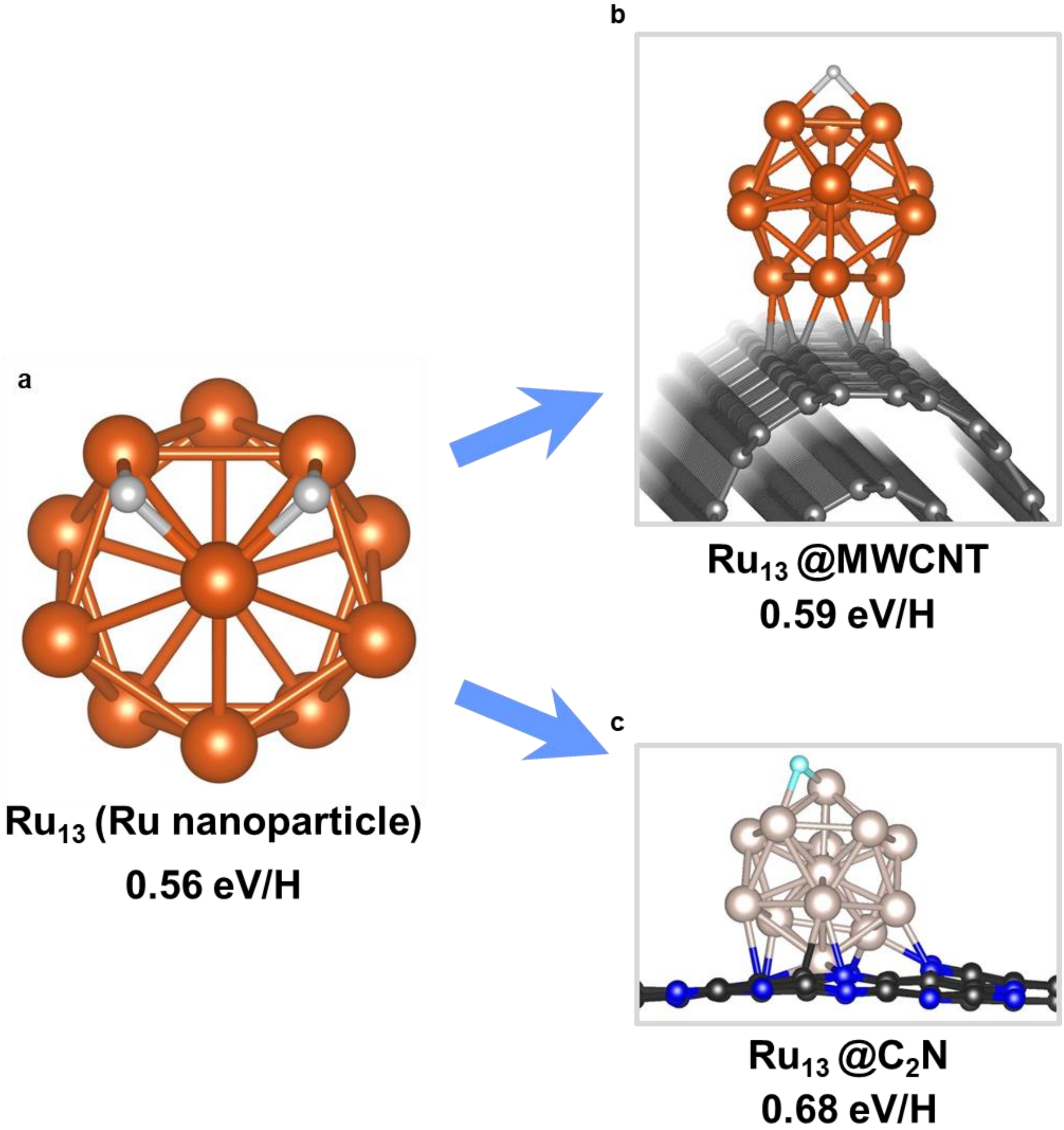

Supplementary Fig. 14 | Hydrogen adsorption configuration on the surfaces of model catalysts and their Ru-H binding energies: **a,** Ru_{13}, **b,** Ru_{13}@MWCNT, **c,** Ru_{13}@C_2N.

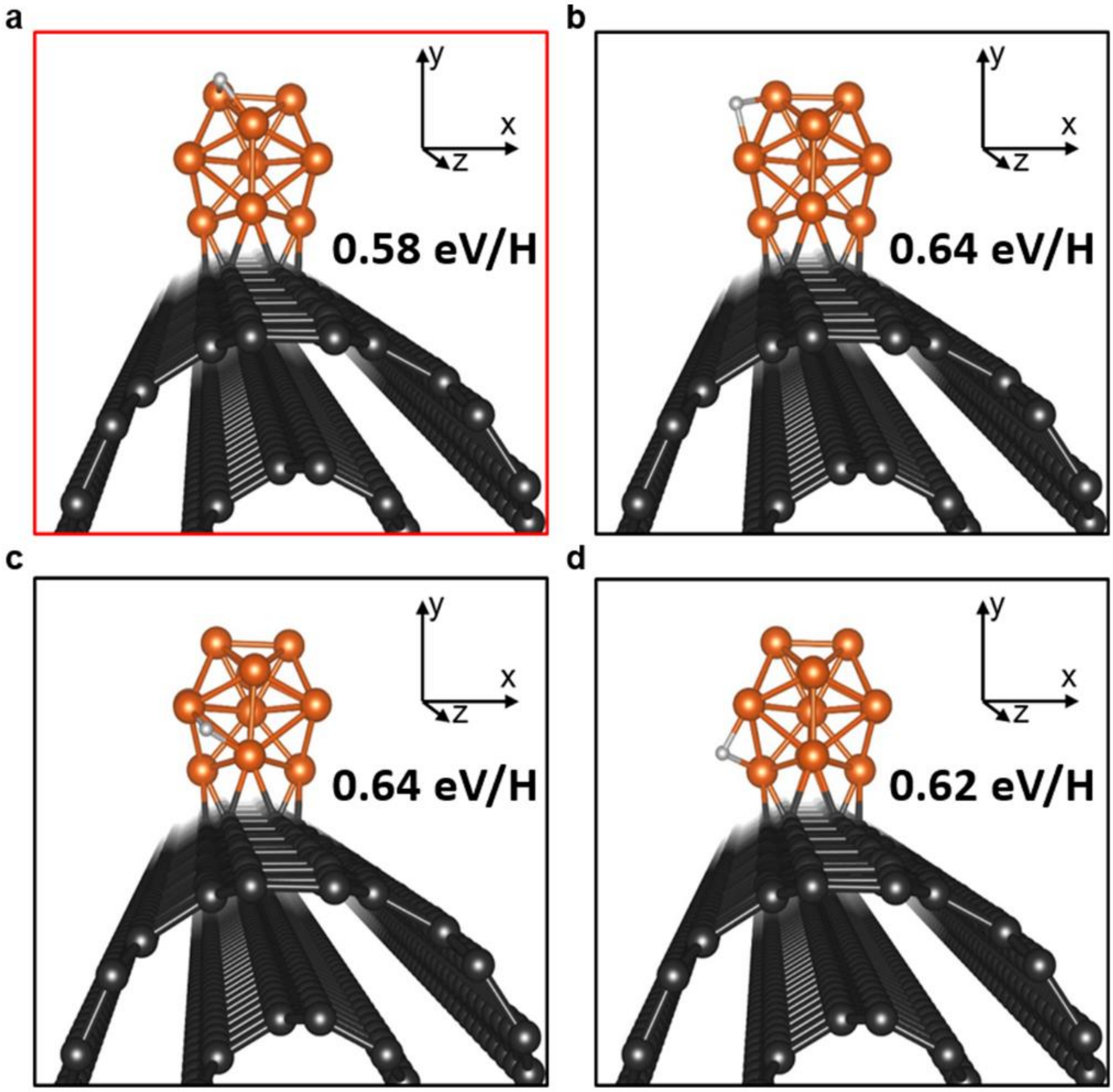

Supplementary Fig. 15 | Hydrogen adsorption configuration at different Ru-H bonding sites on the surface of Ru@MWNCT. **a,** 0.58 eV/H, **b,** 0.64 eV/H, **c,** 0.64 eV/H, **d,** 0.62 eV/H.

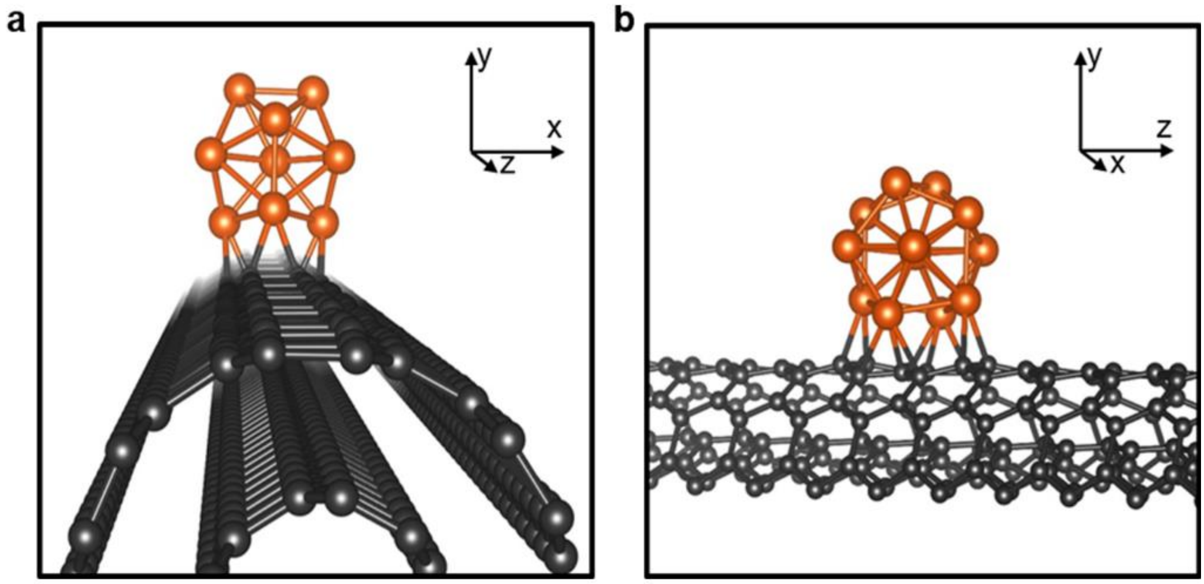

Supplementary Fig. 16 | Formation energy of Ru nanoparticles on the surface of MWCNT. Ten Ru-C bonds are formed with the release of energy (5.23 eV).

Supplementary Table 1 | Elemental composition of the Ru@MWCNT from elemental analysis

	C (wt%)	H (wt%)	O (wt%)	Ru (wt%)[a]	Total (wt%)
Ru@MWNCT	80.79	0.23	3.71	11.60[a]	100

[a] The value calculated by subtracting the values of the C, O and H contents from the sum total of 100 wt%. This value agrees well with the data obtained from the TGA in air.

Supplementary Table 2 | Summary of recently reported representative HER catalysts in acidic electrolyte

Catalyst	Overpotential @10 mAcm^{-2}	Tafel slope (mV dec^{-1})	Reference
Pt/C	16	28	This work
Ru@MWCNT	13	27	This work
Ru@GnP	13	30	Adv. Mater. 30, 1803676 (2018)
Ir@CON	13.4	27	Adv. Mater. 30, 1870401 (2018)
RuO_2-C (Vulcan)	15	26	Electrochim. Acta 55, 1855–1861 (2010)
Ru_2P@PNC/CC-900	15	28	ACS Appl. Energy Mater. 1, 3143–3150 (2018)
IrNi NCs[a]	19	--	Adv. Funct. Mater. 27, 170886 (2017)
RuP (L-RP)	19	37	Adv. Mater. 30, 1800047 (2018)
PtRu@RFCS	19.7	27.2	Energy Environ. Sci. 11, 1232–1239 (2018)
Rh_2P	20	--	J. Am. Chem. Soc. 139, 5494–5502 (2017)
IrCo-PHNC[a]	21	26.6	Adv. Mater. 29, 1703798, (2017)
Ru@C_2N	22	30	Nat. Nanotechnol. 12, 441–446 (2018)
Ru_2P/RGO	22	29	Chem. Commun. 54, 3343–3346 (2018)
Ru-NGC	25	31	Chem. Commun. 55, 965–968 (2019)
Au@PdAg NRBs	26.2	30	J. Am. Chem. Soc. 138, 1414–1419 (2016)
Ru-CCS	27.3	33	J. Mater. Chem. A 6, 2311–2317 (2018)
hcp-Ru@NC-700	27.5	37	ACS Catal. 8, 5714–5720 (2018)
Ru-NC-700	29	28	Nat. Commun. 10, 631 (2019)
Mo@Ru-3	30.5	36.4	J. Mater. Chem. A 7, 2780–2786 (2019)

CoRu@NC	32	47	Nanotechnology 29, 225403 (2018)
0.27-RuO_2@C	33	53	Nano Energy 55, 49–58 (2019)
IrCoNi-PHNC[a]	33	31.9	Adv. Mater. 29, 1703798, (2017)
A-Ni–C	34	41	Nat. Commun. 7, 10667 (2016)
Ru-GLC	35	46	ACS Appl. Mater. Interfaces 8, 35132–35137 (2016)
Pd@Ru NRs	37	33	ACS Appl. Mater. Interfaces 10, 34147−34152 (2018)
RuP_2@NPC	38	38	Angew. Chem. Int. Ed. 56, 11559–11564 (2017)
Ru^0/TiO_2	41	52	J. Colloid Interface Sci. 531, 570–577 (2018)
Ru/CeO_2	47	41	ACS Appl. Mater. Interfaces 10, 6299–6308 (2018)
CoPs nanoplate	48	57	Nat. Mater. 14, 1245–1251 (2015)
$Ni_{1.5}Co_{1.4}P$@Ru	49	49	Chem. Commun. 53, 13153–13156 (2017)
NiRu@N−C (S-2)	50	36	J. Mater. Chem. A 6, 1376−1381 (2018)
Ni@Ni_2P−Ru	51	35	J. Am. Chem. Soc. 140, 2731−2734 (2018)
RuPx@NPC	51	46	ChemSusChem 11, 743–752 (2018)
Pt-MoS_2	53	40	Nat. Commun. 4, 1444 (2013)
Ru-MoO_2	55	44	J. Mater. Chem. A 5, 5475–5485 (2017)
Pt@NHPCP	57	27	Nano Energy 40, 88–94, (2017)
Ru@NG-4[b]	60	41	Sustain. Energy Fuels 1, 1028−1033 (2017)
Ru-HPC	61.6	66.8	Nano Energy 58, 1–10 (2019)
CoS|P/CNT	64	55	Nat. Commun. 7, 10771 (2016)
$MoS_2/CoSe_2$	68	36	Nat. Commun. 6, 5982 (2015)
s-RuS_2/S-rGO	69	64	ACS Appl. Mater. Interfaces 10, 34098−34107 (2018)

$Ru/C_3N_4/C$	70	--	J. Am. Chem. Soc. 138, 16174–16181 (2016)
$WO_{2.9}$	70	50	Nat. Commun. 6, 8064 (2015)
Ni-doped RuO_2 NWs	78	--	J. Mater. Chem. A 7, 6411−6416 (2019)
GCE-S-GNs-1000-CB-Ru	80	61	Carbon 93, 762–773 (2015)
Ru-GC (Ru-MeOH/THF)	83	46	Chem. Commun. 53, 11713–11716 (2017)
W + Ru/C	85	46	ACS Appl. Mater. Interfaces 10, 6354–6360 (2018)
Te@Ru	86	36	Chem. Commun. 55, 1490–1493 (2019)
Ru-GC	90	33	Electrochim. Acta 167, 455–469 (2015)
1D-RuO_2-CN_x	93	40	ACS Appl. Mater. Interfaces 8, 28678−28688 (2016)
$Ru/MoS_2/CP$	96	--	Nanoscale 9, 16616–16621 (2017)
$Ni@Ni_2P$-Ru	99	35	J. Am. Chem. Soc. 140, 2731 (2018)
Ru@CN	126	--	Energy Environ. Sci. 11, 800–806 (2018)
$Cu_{2-x}S$@Ru NPs	129	51	Small 2017, 13, 1700052.
CoN_x/C	133	57	Nat. Commun. 6, 7992 (2015)
MoC_x nano-octahedrons	142	53	Nat. Commun. 6, 6512 (2015)
Co-NG	147	82	Nat. Commun. 6, 8668 (2015)
Edge-terminated MoS_2	149	49	Nat. Commun. 6, 7493 (2015)
SV-MoS_2	170	60	Nat. Mater. 15, 48–53 (2016)
M-MoS_2	175	41	Nat. Commun. 7, 10672 (2016)
$[Mo_3S_{13}]^{2-}$ clusters	180	40	Nat. Chem. 6, 248–253 (2014)
Mesoporous MoS_2	233	50	Nat. Mater. 11, 963–969 (2012)
Exfoliated WS_2 nanosheets	234	55	Nat. Mater. 12, 850–855 (2013)

C_3N_4@NG	240	51.5	Nat. Commun. 5, 3783 (2014)

[a] The acidic electrolyte is 0.1 M aq. $HClO_4$.

[b] The acidic electrolyte is 1.0 M aq. H_2SO_4.

Supplementary Table 3 | Summary of recently reported representative HER catalysts in alkaline electrolyte

Catalyst	Overpotential @10 mAcm^{-2}	Tafel slope (mV dec^{-1})	Reference
Pt/C	33	43	This work
Ru@MWCNT	17	27	This work
Ru/NG-750	8	30	ACS Appl. Mater. Interfaces 9, 3785–3791 (2017)
NiCoMo/Ru-GN	11.4	38	J. Phys. Chem. C 122, 17621–17631 (2018)
Ru-NC-700	12	--	Nat. Commun. 10, 631 (2019)
Ir@CON	12.9	29	Adv. Mater. 30, 1870401 (2018)
Co-substituted Ru	13	29	Nat. Commun. 9, 4958 (2018)
Ru/MoS_2/CP	13	60	Nanoscale 9, 16616–16621 (2017)
Ru_2P/RGO	13	56	Chem. Commun. 54, 3343–3346 (2018)
Ru/C-300	14	32.5	J. Mater. Chem. A 6, 14380–14386 (2018)
Ru/CN-800	14	30	ACS Sustainable Chem. Eng. 6, 11487–11492 (2018)
Ru/3DNPC-500	15	31	ACS Sustainable Chem. Eng. 7, 1178–1184 (2019)
Ru@C_2N	17	38	Nat. Nanotechnol. 12, 441–446 (2017)
RuP (L-RP)	18	34	Adv. Mater. 30, 1800047 (2018)
0.27-RuO_2@C	20	46	Nano Energy 55, 49–58 (2019)
Ru/NC	21	31	J. Mater. Chem. A. 5, 25314–25318 (2017)
Ru@GnP	22	28	Adv. Mater. 30, 1803676 (2018)
4H/fcc Ru NTs	23	29.4	Small 14, 1801090 (2018)

Ru/C	24	33	Adv. Energy Mater. 8, 1801698 (2018)
PP-Ru/RuO_2-GC	25	65	ACS Catal. 8, 11094–11102 (2018)
s-RuS_2/S-rGO	25	29	ACS Appl. Mater. Interfaces 10, 34098−34107 (2018)
Ru@NC	26	36	Angew. Chem. Int. Ed. 130, 5950–5954 (2018)
Cu-doped Ru-RuO_2/C	28	35	Small 14, 1803009 (2018)
RuCo@NC	28	31	Nat. Commun. 8, 14969 (2017)
S-400-H	28	35	Small 14, 1803009 (2018)
NiFeRu-LDH	29	31	Adv. Mater. 30, 1706279 (2018)
Ru-MoO_2	29	31	J. Mater. Chem. A 5, 5475–5485 (2017)
Pd@Ru NRs	30	30	ACS Appl. Mater. Interfaces 10, 34147−34152 (2018)
Ru-Ni@Ni_2P-HNRs	31	41	J. Am. Chem. Soc. 140, 2731–2734 (2018)
NiRu@N−C (S-2)	32	64	J. Mater. Chem. A 6, 1376−1381 (2018)
Ru@CN	32	53	Energy Environ. Sci. 11, 800–806 (2018)
Ru-NGC	37	40	Chem. Commun. 55, 965–968 (2019)
CN_x@Ru/MWCNT	39	28	ChemCatChem 11, 1970–1976 (2019)
Ru@NG-4	40	76	Sustain. Energy Fuels 1, 1028−1033 (2017)
Ru_2Ni_2 SNs	40	23.4	Nano Energy 47, 1−7 (2018)
RuO_2/N−C	40	44	ACS Sustainable Chem. Eng. 6, 11529− 11535 (2018)
Ru ND/C	43.4	49	Chem. Commun. 54, 4613− 4616 (2018)
CoRu@NC	45	66	Nanotechnology 29, 225403 (2018)
MoP/SNG	49	31	ACS Catal. 7, 3030–3038 (2017)

Ru_2P@PNC/CC-900	50	66	ACS Appl. Energy Mater. 1, 3143–3150 (2018)
$Ni_{1.5}Co_{1.4}P$@Ru	52	42	Chem. Commun. 53, 13153–13156 (2017)
Ni-doped RuO_2 NWs	52	--	J. Mater. Chem. A 7, 6411−6416 (2019)
RuP_2@NPC	52	69	Angew. Chem. Int. Ed. 56, 11559–11564 (2017)
hydrous RuO_2	60	--	Chem. Phys. Lett. 673, 89– 92 (2017)
Sr_2RuO_4	61	51	Nat. Commun. 10, 149 (2019)
ah-RuO_2@C	63	62	Nano Energy 55, 49–58 (2019)
crystalline RuO_2	74	--	Chem. Phys. Lett. 673, 89– 92 (2017)
RuP_x@NPC	74	70	ChemSusChem 11, 743–752 (2018)
CoP/CNTs	76	57	Adv. Funct. Mater. 27, 1606635 (2017)
Ru/C_3N_4/C 0.1 M KOH[a]	79	--	J. Am. Chem. Soc. 138, 16174–16181 (2016)
$Ni(OH)_2/MoS_2$	80	60	Nano Energy 37, 74–80 (2017)
$Cu_{2-x}S$@Ru NPs	82	48	Small 13, 1700052 (2017)
NiO/Ni-CNT	86	82	Nat. Commun. 5, 4695 (2014)
$NiFeO_x$/CFP	88	150	Nat. Commun. 6, 7261 (2015)
h-β-Mo_2C@BCN	92	55	J. Mater. Chem. A, 5, 13122-13129 (2017)
1D-RuO_2-CN_x 0.5M KOH[b]	93	40	ACS Appl. Mater. Interfaces 8, 28678-28688 (2016)
RuO_2-NWs@g-CN	95	70	ACS Appl. Mater. Interfaces 8, 28678–28688 (2016)
$NiMoS_2$	98	60	Energy Environ. Sci, 9, 2789–2793 (2016)
$Co(S_xSe_{1-x})_2$	145	85.7	Adv. Funct. Mater. 27, 1701008 (2017)
MoC_x nano-octahedrons	151	59	Nat. Commm. 6, 6512 (2015)
$Co(OH)_2$/Pt(111)	248	--	Nat. Mater. 11, 550–557 (2012).

[a] The alkaline electrolyte is 0.1 M aq. KOH.

[b] The alkaline electrolyte is 0.5 M aq. KOH.

Supplementary Table 4 | Comparison of turnover frequencies (TOFs) of some of recently reported HER catalysts in acidic condition

Catalyst	Overpotential (V)	TOF per active site (H_2 S^{-1})	Reference
Pt/C	0.025	0.67	This work
Ru@MWCNT	0.025	0.70	This work
Ir@CON	0.025	0.66	Adv. Mater. 30, 1803676 (2018)
Ru@C_2N	0.025	0.67	Nat. Nanotechnol. 12, 441-446 (2017)
UHV MoS_2@Au(111)	0.1	1	Science 317, 100-102 (2007)
CoN_x/C	0.1	0.39	Nat. Commun, 6, 7992 (2015)
Ni_2P NPs	0.1	0.015	J. Am. Chem. Soc. 135, 9267-9370 (2013)
CoP NPs	0.1	0.046	Angew. Chem. Int. Ed. 53, 5427-5430 (2014)
Ni_5P_4 (pellet)	0.1	3.5	Energy Environ. Sci. 8, 1027-1034 (2015)
$[Mo_3S_{13}]^{2-}$ cluster	0.2	3	Nat. Chem. 6, 248-253 (2014)
Ni_2P (pellet)	0.2	0.064	Energy Environ. Sci. 8, 1027-1034 (2015)
Ru/g-C_3N_4/C	0.1	4.85	J. Am. Chem. Soc. 138, 16174–16181 (2016)
Ru-CCS	0.05	3.7	J. Mater. Chem. A 6, 2311–2317 (2018)
Ru-NGC	0.03	0.68	Chem. Commun. 55, 965–968 (2019)
Ru@GnP	0.1	0.26	Adv. Mater. 30, 1803676 (2018)
Ru-HPC	0.025	0.18	Nano Energy 58, 1–10 (2019)
RuCeO_2	0.027	0.8	ACS Appl. Mater. Interfaces 10, 6299–6308 (2018)
Ru-GC (Ru-MeOH/THF)	0.1	0.87	Chem. Commun. 53, 11713–11716 (2017)

Te@Ru	0.1	0.82	Chem. Commun. 55, 1490–1493 (2019)
Ru-Ni@Ni_2P-HNRs	0.1	1.1	J. Am. Chem. Soc. 140, 2731–2734 (2018)

Supplementary Table 5 | Comparison of turnover frequencies (TOFs) of some of recently reported HER catalysts in alkaline condition

**Catalyst**	**Overpotential (V)**	**TOF per active site (H_2 S^{-1})**	**Reference**
Pt/C	0.025	0.25	This work
Ru@MWCNT	0.025	0.40	This work
Ir@CON	0.025	0.20	Adv. Mater. 30, 1803676 (2018)
Ru@C_2N	0.025	0.76	Nat. Nanotechnol. 12, 441-446 (2017)
Ni-Mo nanopowder	0.2	0.36	ACS Catal. 3, 166-169 (2013)
Ni_5P_4 (pellet)	0.1	0.79	Energy Environ. Sci. 8, 1027-1034 (2015)
Ni_2P (pellet)	0.2	0.014	Energy Environ. Sci. 8, 1027-1034 (2015)
γ-Mo_2N	0.25	0.07	J. Mater. Chem. A. 3, 8360-8368 (2015)
α-Mo_2C	0.2	0.91	J. Mater. Chem. A. 3, 8360-8368 (2015)
Ru/NG-750	0.1	0.35	ACS Appl. Mater. Interfaces 9, 3785–3791 (2017)
Ru@GnP	0.1	0.145	Adv. Mater. 30, 1803676 (2018)
Ru/C	0.04	0.18	Adv. Energy Mater. 8, 1801698 (2018)

Supplementary Table 6 | Amount of hydrogen production per voltage applied for samples (constant current = 10 mA)*

Time (h)	Carbon paper (μmol V^{-1})	Pt/C (μmol V^{-1})	Ru@MWCNT (μmol V^{-1})
1	58.62 ± 2.84	94.69 ± 2.36	109.86 ± 1.86
2	118.81 ± 3.79	190.04 ± 1.99	221.07 ± 1.28
3	178.76 ± 3.34	286.41 ± 1.93	332.93 ± 1.92
4	239.03 ± 3.99	382.19 ± 1.44	444.12 ± 2.03
5	298.81 ± 4.43	478.00 ± 2.23	555.00 ± 3.82
6	358.99 ± 4.47	574.65 ± 2.81	665.93 ± 4.37
7	419.13 ± 4.56	671.21 ± 2.51	776.54 ± 4.54
8	479.09 ± 4.56	766.62 ± 4.01	887.58 ± 5.56
9	539.07 ± 3.75	862.42 ± 3.22	998.69 ± 5.01
10	604.50 ± 12.74	958.23 ± 3.01	1109.13 ± 6.27
11	664.69 ± 13.19	1054.15 ± 3.20	1220.43 ± 6.08
12	725.48 ± 13.53	1149.96 ± 4.34	1331.09 ± 5.49
13	786.15 ± 13.12	1245.33 ± 4.00	1442.64 ± 5.44
14	846.15 ± 12.72	1341.16 ± 2.92	1553.88 ± 6.03
15	906.58 ± 12.13	1437.30 ± 2.82	1664.99 ± 6.38
16	966.78 ± 11.98	1532.24 ± 1.58	1776.47 ± 5.99
17	1026.88 ± 11.95	1627.91 ± 2.00	1887.83 ± 5.78
18	1087.40 ± 12.97	1724.70 ± 2.15	1999.47 ± 5.94
19	1147.51 ± 13.12	1820.02 ± 1.79	2110.64 ± 4.53
20	1208.21 ± 12.43	1915.34 ± 0.73	2222.33 ± 5.61

* This value is the average of three different electrode test results.

Supplementary Table 7 | Amount of hydrogen production per voltage applied for samples (constant current = 15 mA)*

Time (h)	Carbon paper (μmol V^{-1})	Pt/C (μmol V^{-1})	Ru@MWCNT (μmol V^{-1})
1	88.72 ± 1.35	141.80 ± 0.23	161.90 ± 0.95
2	177.89 ± 2.85	283.40 ± 1.38	322.63 ± 0.63
3	267.26 ± 3.43	425.49 ± 1.37	483.40 ± 0.83
4	355.95 ± 3.79	567.45 ± 1.28	644.49 ± 0.70
5	445.24 ± 3.82	709.31 ± 1.55	805.83 ± 0.35
6	534.20 ± 3.67	851.17 ± 0.97	967.09 ± 0.47
7	623.68 ± 4.24	992.88 ± 1.94	1127.44 ± 1.33
8	712.84 ± 4.51	1134.87 ± 1.87	1288.50 ± 1.53
9	802.39 ± 4.30	1277.40 ± 2.14	1450.61 ± 1.85
10	891.55 ± 4.18	1419.64 ± 1.02	1611.35 ± 2.33
11	980.27 ± 5.21	1561.21 ± 0.77	1772.34 ± 2.86
12	1070.19 ± 6.13	1702.65 ± 1.58	1933.45 ± 2.46
13	1159.70 ± 6.30	1844.63 ± 2.29	2094.48 ± 1.60
14	1248.95 ± 6.01	1986.47 ± 3.18	2254.78 ± 1.28
15	1337.96 ± 5.37	2128.19 ± 3.11	2416.54 ± 2.15
16	1427.94 ± 4.63	2269.44 ± 3.48	2578.66 ± 1.73
17	1517.88 ± 5.08	2411.71 ± 3.66	2739.58 ± 2.27
18	1606.86 ± 5.67	2552.85 ± 3.78	2900.39 ± 3.06
19	1695.99 ± 5.11	2694.25 ± 4.41	3061.15 ± 2.49
20	1785.27 ± 5.00	2835.72 ± 3.68	3221.92 ± 2.41

* This value is the average of three different electrode test results.

Supplementary Table 8 | Amount of hydrogen production per voltage applied for samples (constant current = 20 mA)*

Time (h)	Carbon paper (μmol V^{-1})	Pt/C (μmol V^{-1})	Ru@MWCNT (μmol V^{-1})
1	116.38 ± 0.45	178.77 ± 1.57	209.38 ± 2.34
2	232.60 ± 1.57	357.40 ± 1.39	418.50 ± 3.87
3	348.69 ± 2.28	536.57 ± 1.37	628.05 ± 5.49
4	465.04 ± 3.37	715.65 ± 0.61	838.08 ± 7.70
5	581.66 ± 3.40	893.75 ± 2.72	1047.46 ± 9.17
6	697.66 ± 4.12	1073.02 ± 2.72	1256.78 ± 10.48
7	813.88 ± 4.94	1252.58 ± 2.77	1467.55 ± 12.45
8	929.97 ± 5.88	1431.53 ± 3.26	1677.55 ± 13.69
9	1046.43 ± 7.13	1610.02 ± 3.41	1886.91 ± 17.16
10	1162.66 ± 8.03	1790.15 ± 4.39	2096.83 ± 17.78
11	1279.90 ± 9.64	1969.88 ± 3.72	2306.46 ± 21.32
12	1396.34 ± 9.80	2148.44 ± 3.52	2517.04 ± 22.03
13	1513.37 ± 10.35	2328.40 ± 3.55	2726.40 ± 24.20
14	1629.91 ± 11.80	2507.83 ± 4.42	2935.24 ± 26.28
15	1746.72 ± 13.52	2687.14 ± 3.43	3144.61 ± 28.59
16	1862.97 ± 14.82	2865.93 ± 3.12	3353.40 ± 30.85
17	1979.57 ± 16.11	3044.55 ± 3.42	3564.19 ± 33.59
18	2096.31 ± 15.81	3222.43 ± 3.69	3774.11 ± 35.69
19	2212.74 ± 16.61	3401.11 ± 4.37	3983.92 ± 37.85
20	2329.00 ± 17.64	3579.87 ± 4.75	4193.98 ± 39.75

* This value is the average of three different electrode test results.

Supplementary Table 9 | Hydrogen production per power consumption and Faradaic efficiency of samples*

Sample	Hydrogen production per power consumption (L kWh^{-1})	Faradaic efficiency (%)
Carbon paper	135.31 ± 1.92 (@ 10 mA)<br>131.93 ± 0.86 (@ 15 mA)<br>135.22 ± 0.91 (@ 20 mA)	11.39 ± 0.24 (@ 1.8 V)
Pt/C	214.55 ± 0.79 (@ 10 mA)<br>211.90 ± 0.30 (@ 15 mA)<br>200.63 ± 0.40 (@ 20 mA)	46.99 ± 7.12 (@ 1.5 V)<br>81.98 ± 4.66 (@ 1.6 V)<br>85.88 ± 0.34 (@ 1.7 V)<br>85.97 ± 0.14 (@ 1.8 V)
Ru@MWCNT	247.65 ± 1.16 (@ 10 mA)<br>240.84 ± 0.13 (@ 15 mA)<br>234.96 ± 2.13 (@ 20 mA)	85.88 ± 3.99 (@ 1.5 V)<br>87.31 ± 0.78 (@ 1.6 V)<br>92.24 ± 1.28 (@ 1.7 V)<br>92.28 ± 0.11 (@ 1.8 V)

* This value is the average of three different electrode test results.
