# Supplementary material for: Ruthenium anchored on carbon nanotube electrocatalyst for hydrogen production with enhanced Faradaic efficiency
Source: Nat Commun. 2020 Mar 9;11:1278. doi: 10.1038/s41467-020-15069-3 (PMC7062887; doi:10.1038/s41467-020-15069-3)
Supplement: Supplementary file 4 — Description of additional supplementary files [file 41467_2020_15069_MOESM4_ESM.docx]

Description of Additional Supplementary Files

File Name: Supplementary Video 1

Description: An actual water splitting device consists of carbon paper electrodes coated with Ru@MWCNT as a HER catalyst and IrO_2_ as an OER catalyst, respectively. Another device shows mesh-type titanium electrodes coated with Ru@MWCNT and IrO_2_ at operating voltages of 1.6V and 1.8V.
